# The role of women's leadership and gender equity in leadership and health system strengthening

**DOI:** 10.1017/gheg.2016.22

**Published:** 2017-05-17

**Authors:** R. Dhatt, S. Theobald, S. Buzuzi, B. Ros, S. Vong, K. Muraya, S. Molyneux, K. Hawkins, C. González-Beiras, K. Ronsin, D. Lichtenstein, K. Wilkins, K. Thompson, K. Davis, C. Jackson

**Affiliations:** 1 Women in Global Health, 30901 Wiegmen Road, Hayward, CA 94544, USA; 2 Liverpool School of Tropical Medicine, Pembroke Pl, Liverpool L3 5QA, UK; 3 Institute of Development Studies, Institute of Development Studies, Library Road Brighton BN1 9RE, UK; 4 Biomedical Training Research Institute, 10 Seagrave Road, Avondale Harare, Zimbabwe; 5 Cambodia Development Resource Institute, 56 St. 315, Phnom Penh 622, Cambodia; 6 Kenya Medical Research Institute – KEMRI-Wellcome Trust Research Programme, P.O. Box 230, Kilifi, Kenya; 7 Nuffield Department of Medicine, Centre for Global Health and Tropical Medicine, University of Oxford, Oxford, UK; 8 Pamoja Communications, UK Bishopstone, 36 Crescent Road, Worthing BN11 1RL, UK; 9 Institute of Hygiene and Tropical Medicine, NOVA University of Lisbon, Rua da Junqueira 100, Lisbon, Portugal

**Keywords:** Gender and health systems, gender equality, gender in health systems resilience, gender responsive, global health, health systems, health systems strengthening, international health, women in global health, women leadership in health, women's leadership, women's leadership in global health

## Abstract

Gender equity is imperative to the attainment of healthy lives and wellbeing of all, and promoting gender equity in leadership in the health sector is an important part of this endeavour. This empirical research examines gender and leadership in the health sector, pooling learning from three complementary data sources: literature review, quantitative analysis of gender and leadership positions in global health organisations and qualitative life histories with health workers in Cambodia, Kenya and Zimbabwe. The findings highlight gender biases in leadership in global health, with women underrepresented. Gender roles, relations, norms and expectations shape progression and leadership at multiple levels. Increasing women's leadership within global health is an opportunity to further health system resilience and system responsiveness. We conclude with an agenda and tangible next steps of action for promoting women's leadership in health as a means to promote the global goals of achieving gender equity.

## Introduction

The Sustainable Development Goals (SDGs) provide a normative global vision for worldwide social improvements and progress [1]. Central to achieving these goals is continued progress in promoting healthy lives and wellbeing for all people, especially marginalised and vulnerable populations [2]; progress can be hindered by conditions of inequity [3, 4]. The SDGs also provide guidance on global efforts to improve gender equity, with targets that relate to ending gender-based discrimination and resource allocation, recognising and supporting women in leadership positions, addressing the issue of unpaid care, stopping harmful and unhealthy practices, improving sexual and reproductive health and strengthening policy in these areas. In practice, the endeavour of building healthy communities is enmeshed with efforts to overcome inequity.

Demographic, geographic and epidemiological change means that the burden of disease felt by women is significantly changing in scope [5], with ‘chronic diseases and NCDs such as cardiovascular disorders, stroke, cancer, diabetes, chronic obstructive pulmonary disease and mental health disorders now the leading causes of death and disability for women in almost all countries’ [6]. As health issues evolve, so must our response, within every area of global health and development [7, 8]. Governance is a core pillar of health systems and greater parity and gender responsive, transformative leadership are essential in our efforts to strengthen health systems and meet the gender- and health-related SDGs [9, 10].

Women make up the vast majority of those working in the field of global health; however, they are underrepresented within top institutions, in global policy and governance forums, in thought leadership panels, and across decision-making structures in the public and private sectors [11]. While gender parity in leadership has not been achieved in many fields – including business, law, science, education, technology and political space – the gender gap in global health is particularly problematic as it is not reflective of the practices and goals within the sector [12]. Without the full and equal participation of all relevant stakeholders striving to achieve the global priorities, the global community is denying itself a valuable resource in reaching ambitious goals. It is important to recognise that the detrimental health impact of a gender imbalanced global health leadership remains largely unknown, while the benefits of gender equal leadership are many [13].

This paper presents a partnership between Women in Global Health (WGH)†^1^ and the Research in Gender and Ethics (RinGS)^2^ consortium to conduct empirical research to examine the realities, challenges and opportunities of women's leadership in global health internationally and within Cambodia, Zimbabwe and Kenya and the implications for the creation of stronger and fairer health systems. It points to deficiencies in this area and signposts ways in which they could be overcome.

## Methodology

### Setting the context

This multi-method empirical research combines learning from three complementary data sources to assess gender equity, women's leadership and health systems strengthening, as discussed below:

1. Data source 1: Literature review

We conducted an extensive literature review using the US National Library of Medicine with the terminology ‘[women in global health], [women leadership in global health], [women in health], [women leadership in health], [women leadership in medicine], [gender in health systems resilience], [gender and health systems].’ This was followed by grey literature review retrieved in electronic format from government, international agencies, academics, conference publications and health industries websites from January to May 2016.

1. Data source 2: Quantitative analysis of gender and leadership positions in global health organisations

We researched primary quantitative data on the relationship between gender and leadership positions in Global Health Organisations and institutions through analysing data from purposefully selected international organisations working within global health. We used the public directories of conference programmes, leadership in ranked global health organisations and elected officials within ministries of health to gather data on the proportion of male and female leaders.

1. Data source 3: Qualitative life histories with health workers in Cambodia, Kenya and Zimbabwe

Given the paucity of data on women and leadership in the health sector, especially in low- and middle-income countries, we partnered with RinGs^3^ to synthesise findings from three qualitative research studies that aimed to explore how gender roles and relations impact on leadership in the health sector in different resource poor contexts as follows: Cambodia (Vong and Ros), Zimbabwe (Buzuzi *et al*.) and Kenya (Muraya *et al*.). For the sake of comparability we have only used findings derived from the life histories components of the research. There has been a growing interest and use of life histories – especially amongst researchers influenced by feminist epistemologies – to capture experiences and perceptions of research participants that may have been unheard and/or need situating within a particular context [14]. Life histories are arguably particularly conducive to gender analysis as participants are enabled to narrate in their own voices their experiences of work and how gender shaped their experiences [14].

### Sampling and process of data collection

The Cambodian study took place in one province, covering two operational districts; the Zimbabwean study was undertaken in four districts in the Midlands Province and in the Kenya study in Mombasa and Kilifi counties in the coastal region. Life histories were conducted with purposively selected cadres of women and men health workers to capture diversity in terms of hierarchy, experience, age and gender. In Cambodia, 20 life histories were conducted with 16 women and four men, in Zimbabwe, 19, with 11 women and eight men and in Kenya 25, with 12 women and 13 men. Mindful of health worker busy workloads, researchers negotiated a time and a place to conduct the life histories, which was most convenient for participants. Life histories were carried out by experienced social science researchers with the aim of enabling the participants to feel at ease, demarcate the stages of their life and career progression in their own terms and enable a reflective discussion on gender, expectations and roles and implications for access to training, promotion and career advancement opportunities. There was a focus through time (from childhood to training to employment) and also on the ways in which factors at different levels shape experiences.

### Analysis

All life history interviews were recorded (following informed consent), transcribed and analysed inductively following the framework approach to enable the identification of key themes emerging from the data with a particular focus on gender roles and relations [15].

### Ethics

Informed consent was sought from all participants, and researchers were particularly careful to develop a good rapport with participants to facilitate a reflective and open discussion. Given small numbers of health workers in some contexts, particular care was taken to ensure confidentiality in the write up and dissemination of findings. Ethical clearance was gained from the respective national ethical boards.

As the concepts detailed in this paper use terminology that may be interpreted differently based on one's background or discipline, we make explicit our definitions as follows in Box 1, ‘Selected Terminology’.

Box 1. Selected terminology.

The concepts detailed in this paper use terminology that may be interpreted differently based on one's background or discipline. Hence, we make explicit our definitions as follows:

Global health

Global Health is the multi-disciplinary field of service, research, and training that seeks to improve the health of both individuals and populations and to achieve health equity for all people worldwide, especially for the resource-poor [16]. It ‘stands for a new context, a new awareness, a new strategic approach to matters of international health’ [17].

Gender v. sex

Gender – the socially constructed roles, behaviours, activities and attributes that a given society considers appropriate for males, females and other genders – affects how people live, work and relate to each other at all levels, including in relation to the health system [18] whereas sex encompasses the biological differences between male and female, such as chromosomes and reproductive systems. Gender roles vary over time and between cultures, but sex varies little [18].

Gender responsive

Being gender-responsive means recognising and understanding the lives, roles and contribution of women and men, ensuring that women benefit equally from interventions [19, 20].

Gender mainstreaming

Gender mainstreaming aims to promote gender equality. It is a process of analysing the consequences for women and men of any planned action, such as programming or policy, ensuring that the perspectives of both men and women are present in design, implementation, monitoring and evaluation [21]. Gender mainstreaming always includes a systematic gender analysis, analysing the situation of women, men, boys and girls and their relationships to each other. Gender should be integrated in every aspect of programming and policy development, and actions working towards gender equality should be prioritised [22].

Gender equity

Equity can be distinguished from equality in that *while equality carries some notion of ‘sameness’, equity carries some notion of ‘fairness’*. Therefore while a focus on equality would argue that men and women should be treated exactly the same (that is, not discriminated against in the provision of healthcare explicitly on the basis of their sex), a focus on gender equity argues that men and women may have different needs and face different barriers to meeting those needs or having them met (Gender and Health Group, 1999) [23].

Gender equality in global health leadership

Having gender equality in global health leadership refers to women and men having equal access to leadership positions, without norms, prejudices, discrimination, legislature or other standing in the way. Equal leadership means equal representation, power, rights and influence between genders. One way of monitoring this is by comparing the number of men and women in leadership positions, gender equality referring to at least 40% and not more than 60% of one gender. However, equal representation does not necessarily mean equal impact.

In the same way as gender is a social variable, there are other social variables influencing people's position in society; ethnicity, religion, class, age, disability, sexual orientation among others. Intersectionality refers to the fact that these variables interact, ensuring some people privileges and making it easier for them to reach global health leadership positions, resulting in diversity in the leadership not reaching its full potential.

Gender-responsive, transformative leadership

Gender-responsive, transformative leadership is about approaching leadership through a multi-disciplinary value based approach. Gender-responsive leadership strives to gender mainstream ‘the process of assessing the implications for women and men of any planned action, including legislation, policies or programmes, in all areas and at all levels. It is a strategy for making women's as well as men's concerns and experiences an integral dimension of the design, implementation, monitoring and evaluation of policies and programmes in all political, economic and societal spheres, so that women and men benefit equally and inequality is not perpetrated’ [22]. Gender-responsive, transformative leadership is not only about achieving gender equality, but about equipping women and men with the tools to change the mindset of society in manner to achieve gender equity at all levels. In the global health community, using such an approach to leadership can be a tool to achieve greater gender equity for achieving health and wellbeing of all people [22].

Health system strengthening and resilience

A Health system consists of all people, institutions, resources and activities whose primary purpose is to promote, restore and maintain health [8]. Health system strengthening and resilience involves integrating national policies, strategies and plans with evidence-based research to evaluate and develop a health care system, which meets the needs of the community, while being responsive to disasters or public health crises [24, 25]. It involves shifting the focus from responding to singular diseases, to considering the broader system as a whole, and its ability to provide for the health care needs of everyone in the community [25]. Women play a critical role in strengthening health systems by giving voice to the concerns of half the population whose health care needs may be unmet and they also provide the bulk of health care worldwide in the formal and informal sectors [9]. In their 2016 Strategic Report, The Alliance for Health Policy and Systems Research emphasised the need to empower more women as leaders in conducting health systems research and policy making, and included them as one of their target groups in Objective 2, ‘Support institutional capacity for the conduct and uptake of health policy and systems research [25].’

## Results

### Data sources 1 & 2: Status of women in global health: learning from the literature and quantitative research

In many countries, more than 75% of people engaged and working in global health are women, but this proportion of women is not reflected at the top levels of leadership [26]. The Lancet Commission on Women and Health revealed that women are contributing around US$3 trillion to global health care, but nearly half of this [2.35% of global gross domestic product (GDP)] is unpaid. The vast contribution of women and the integral role they play as a large part of the health-care labour force is often underappreciated and underrecognised [6]. In a study on the financial value of women's contributions in the health system in 2010, which included the analysis of 32 countries and 52% of the world's population, Langer estimated that the financial value of women's contributions in the health system in 2010 was 2.35% of global GDP for unpaid work (domestic care for family members, officially compensated in a select few countries) and 2.47% of GDP for paid work – the equivalent of US$3.052 trillion [6].

Inequity is widespread, especially at the highest levels of management and leadership, for instance, in 2015, only 27% of Ministers of Health were women [27]. In 2014, only 24% of directors of global health centres at the top 50 US medical schools were women [12]. At the 68th World Health Assembly in May 2015 of the World Health Organization, only 23% of member state delegations had a woman in the role of chief delegate [28].

There are also large discrepancies with the numbers at the top leadership positions in global health-funding agencies (including the Global Fund to fight AIDS, TB and Malaria, GAVI, UNITAID, PEPFAR, PMI, the World Bank and UNAIDS) [29]. The *Secretary General*'*s Report on the Improvement of the Status of Women in the United Nations System* (2014) showed a persistent inverse relationship between level of professional position and female representation [30]. Within the Bill and Melinda Gates Foundation, the executive leadership team was only 25% women [31] and within the United Kingdom's Department for International Development 33% of leadership positions are occupied by women [32].

### Data source 3: Gender equity and women's leadership in human resources for health and health systems at national level: learning from qualitative research

Our analysis from the multiple contexts shows how gender roles, relations, norms and expectations shape progression and leadership at three intersecting levels: at the individual level; within households and communities and within health systems and institutions.

## Gender, motivation and possibilities at the individual level

Gendered motivation and decision making affects the uptake of leadership opportunities at the individual level. In Cambodia women constitute only 20% of those in senior roles in the Ministry of Health. Capacity, determination to succeed, and confidence supported their progression. When offered leadership positions women took a consensual approach to acceptance, seeking approval from families first.

Some female Cambodian managers emphasised ‘thinking like men’ and considered that ‘women can do things like men’ to motivate themselves to take the leadership position.

“*I always perceive that whatever men can do, women can also do it. I always want to show my output and results to others*.” (F, Married, 58_5)

Others mentioned hard work or willingness to try new challenges as a way for women to step into the leadership position.

“*Though I was nervous, I had to do and must achieve it. At that time, I told myself I had to try it!*” (F, Married, 44_11)

## ‘Carrying the baby’: Gendered roles and reproductive responsibilities and their impact on leadership at the community level

Within Zimbabwe family responsibilities variously affected individual men and women with regards to taking up further studies or training opportunities. Female health workers reiterated that such decisions were difficult to make when children were still young and the training compelled them to be away for long periods. Both men and women noted that they considered paying school fees for their children first before they pay for their own studies. However, men tended to ‘*be impatient*’ and pursue self-funding courses, which gave them an advantage over women during interviews for promotion. More women reported that they lost senior positions to men because the men had better qualifications. In Zimbabwe, it is the norm that when husbands relocate for work, wives resign from their jobs to follow them. Through this process they can sacrifice accrued years of service and associated training and promotion opportunities.

In Cambodia women discussed the challenges they faced in juggling and family responsibilities, including breastfeeding, child raising and domestic chores, and their decisions tended to prioritise families rather than their career.

“*The hardest thing for women was when I had meeting at province. I had to bring both my husband and children to go with me. After the meeting, I had to rush to breast-feed my children. If men have mission at province, they will go alone.*” (F, Married, 44_11)

“*…I carried my child with me as no one took care my child. Here [health facility] people can help me took after my child…*” (F, Married, 30_7)

Cambodian women who progressed to leadership levels emphasised the strong family/parental and spousal support in their career, or were single or married late.

Some Cambodian male managers emphasised that women's role and priorities should be in the home. Similarly, analysis of research on gender and leadership in Kenya, suggested that women were often perceived as child-bearers and nurturers and that this was seen as a disadvantage to their career progression and ability to take up health leadership positions. As the following comment from a female senior manager suggests, this view was not only held by men:

“*[When appointing a health manager]…if she is female you have to consider if she has kids or not. That makes a difference. You will find that you select someone, train them and invest so much in them, then after working for only a few months they fall pregnant and go off on maternity leave. Also once they have a child, the women tend to become irregular with work, there isn*'*t that commitment…*” (R016, female senior manager)

## The impact of norms, stereotypes and expectations within institutions on leadership

In Zimbabwe, human resource managers prefer to deploy men to very rural areas as they believe they will stay longer and not request transfers. Rural posting was discussed positively as a way to gain a wide range of experiences (in the absence of senior medical staff) and in turn was valued (by men) in terms of future access to training, invitations to international workshops, and promotion.

Within Kenya professional hierarchies play a role in the appointment of health leaders and this in turn can be shaped by gender; medical doctors (who are often male, although this situation is changing over time) tend to be preferentially selected for leadership positions. As the following quote suggests, these gendered professional hierarchies can influence leadership training and style:

“*A doctor will always get the position…[but]…we need to change our perspective. If people can see nurses and other health professionals can also lead, doctors will also learn*” (R011, male senior manager)

Research in Cambodia showed positive trends in national and provincial government structures in terms of greater sensitivity with the implementation of gender focal points and gender working groups to provide training on gender and leadership skills to health workers and ensure women representation at all levels.

“*I already have a plan to promote women in leadership. First, I will organize training on gender to my staff and monitor their performance… Second, we will build capacity of men and women in leadership skills… I want to see more women to become the head of health centers*” (F, Married, 58_5)

Within provinces and districts the large majority of health workers are female and many women were appreciative of the support they received from (mainly male) superiors in trouble shooting and career progression (Box 2).

Box 2. Community health worker backgrounds.

Additional information for Cambodia

In Cambodia, the study was conducted in two operational districts (Battambang and Moung Russei), in Battambang Province [33]. 20 participants (14 females and six males) were purposively recruited based on four criteria: age, service date, skills and leadership positions. The ages of respondents ranged from 30 to 64 and majority of them is at 50s. Their service dates were drawn between 1980s, 1990s and 2000s. We also selected respondents with different professional skills, ranging from primary to secondary nursing or midwifery, to medical associate or medical doctor. The following leadership cadres of health managers were represented in our respondent selection:

- Deputy of Provincial Health Department
- Head of Operational District
- Deputy of Operational District
- Head of Referral Hospital
- Head of Health Center
- Deputy of Health Center

Additional information for Kenya

There were two case study counties (Kilifi and Mombasa) in Kenya. Respondents were drawn from the various County & Sub-County Health Management Teams within those two counties [34]. Of the 25 respondents, 12 were male and 13 were female. The following cadres of health managers were represented in the respondent selection:

- County health executives
- County directors of health
- County chief officers of health
- Sub-county medical officers of health
- Sub-county public health nurses
- Sub-county facility management nurses
- Sub-county public health officers
- Sub-county disease surveillance coordinators and
- Sub-county programme coordinators

Additional information for Zimbabwe

The Zimbabwean study was undertaken in four districts in the (Kwekwe, Chirumanzu, Gokwe North and Gokwe South) Midlands Province [35]. Life/career posting histories were conducted with purposively selected cadres of women and men health workers to capture diversity in terms of hierarchy, experience, age and gender. In Zimbabwe, 19 life histories were conducted with 11 women and eight men and key informant interviews *N* = 11 (six males, five females) with human resource managers at different levels.

Questionnaires *N* = 140 (57M, 83F) administered to older health workers (28 Environmental Health Technicians/officers, 32 Midwives, 21 Registered General Nurses, 59 State Certified Nurses/Primary Care Nurses). We targeted health workers have been in the health sector since 2000 up to 2009 when the multicurrency system was introduced.

Our key informants included:

- District Human Resources Officers
- District Nursing Officers
- Provincial Health Services Administrator
- Provincial Nursing Officer
- Matrons/Sisters-in-Charge at health facilities and or heads of departments within health facility e.g. Maternal and Child Health

OUTSTANDING

- Human Resources Director

## Discussion

The global health community has recognised the importance of gender equity and its achievement to be imperative to the attainment of healthy lives and wellbeing of all [4, 9]. As a part of the broader development community, the global health community remain the greatest advocates and investors in women and girls [7]. The SDGs have brought new impetus to the need for gender equity and SDG 5: ‘Achieve gender equality and empower all women and girls’ has a specific target on leadership: ‘5.5: Ensure women's full and effective participation and equal opportunities for leadership at all levels of decision-making in political, economic and public life’

The different data sources in this piece all highlight how gender inequity plays out at different levels of the health system across multiple contexts. There are some inspiring exceptions – ‘positive deviants’ – but overall leadership within this feminised sector is very male dominated. So without gender equity, what is the collateral damage? What is the significance of gender bias in health, health systems strengthening, health outcomes and the achieving the SDGs?

The global health community is not systematically addressing its own gender gap in global health leadership [12]. Achieving gender parity in global health leadership at all levels of health systems is fundamental to tapping into all the potential of the global health community and creating solutions, which are both gender responsive and effective. Women's leadership is particularly important in addressing problems that directly affect their own lives, and in addressing areas with increasing inequities [9].

Langer states that currently women struggle to function to their full capacity due in large part to the lack of gender-sensitive policies ‘that enable women to integrate their social, biological and occupational roles.’ [6]. The learning from the qualitative life histories show how gender norms, expectations, roles and responsibilities at the individual level, within household, communities and institutions affect entry to the health sector, progression and leadership. Despite multiple challenges there are some examples of ‘positive deviance’, women who have managed to reach leadership positions. In these cases family and manager support has been very important for professional development. To support positive change requires changes in personal and family attitudes and practices. Institutions in the health system must put in place supportive policies and practices (for example, in relation to recruitment, supervision, strategies to address violence, child care and training). Health system governance should be approached through a gender lens to identify gender-based issues and inequities and lead to the creation of structures that explicitly address these issues.

Current research that has analysed gender inequity in health has shown that the presence of gender inequality damages the physical and mental health of millions of girls and women across the globe. Furthermore, it has detrimental outcomes on the physical and mental health of men and boys, even considering the many ways it benefits men through resources, power, authority and control [4]. These findings create a compelling case regarding the need for gender equality within society and in global health as a service, but further evidence is needed on the impact that gender inequality in global health leadership has on health systems strengthening, its programming and its effectiveness.

Existing research on gender parity in leadership and empowerment of women in many other sectors – science and technology, business and other non-health fields – demonstrates the financial loss of not facilitating conditions for women to be equals in economic participation. McKinsey, a leading consulting group, estimates that achieving gender parity would be worth around US$28 trillion to the global economy, an increase of 26% from levels projected given conditions of continued gender inequity [36]. While a rights-based approach should be reason enough for achieving gender parity in global health leadership, the global health research community must make an evidence-based case for gender parity, based on health outcomes, to support a more expedited transformation of the sector's policies. The global health community must evaluate leadership at all levels and how it impacts on health outcomes.

The evidence is limited but suggests clear links between women's leadership and more equitable health outcomes in different contexts and at different levels of the health system. A review done by Down et al in 2014, using randomised trials, showed that women in leadership positions in governmental organisations implement different policies than men and that these policies are more supportive of women and children [12]. After a 1993 constitutional amendment in India that required rural villages to state whether or not their village was headed by a female leaders, a field study was conducted that looked at female leadership and outcomes, and showed that women tended to invest in public works more closely linked to women's concerns, such as clean drinking water [12]. Men invested in works more aligned with men's concerns and activities, such as irrigation systems for farming. Even more notably, a review ten years later demonstrated that those villages that had female leaders were more likely to create enabling environments for girls – highlighting education, elevated job aspirations and a shift away from an emphasis on domestic chores [12]. In the Downs study, gender was shown to influence decision making and have an impact on women's health in field analysis [12]. Larger scale studies which systematically approach global health leadership through a gender analysis are needed to grasp the full effect of gender inequity in the global health community and create gender-responsive resolutions.

## Time to act: setting an agenda for change

We need to build gender equity in global health leadership at all levels. Our analysis shows how health systems depend on women as providers of health care, yet women rarely lead within the systems they contribute so much to. Where they do lead, they often utilise different styles and set different priorities that are arguably more responsive to health needs of the full spectrum of people – women, men, girls, boys and people of other genders. We need strong responsive health systems and this means making the most of the leadership talent pool. Below, we outline an agenda for action.

The way forward:

1. Leadership that is gender responsive and institutionalised

Gender-responsive leadership is needed at all levels of health systems, as is work towards eliminating gender bias and discrimination [9] in order to support full participation of all genders and assure equitable access to opportunities.

- All people, regardless of gender, working in the global health field and health sector and especially in leadership roles, should be required to go through a gender-responsive training as part of a core competencies training [22].

1. Development of enabling environments for women's leadership

Recognising and increasing the visibility of women's leadership in global health through: (1) hosting gender balanced events; (2) a recognition system; and (3) active recruitment of women leaders at all levels should be a priority in global health [11–13, 26–32]. Such environments will contribute to institutional re-structuring that provides support to women both in building careers and in progressing and continuing to achieve throughout the life course.

- Increase thought leadership events related to women's role in global health [11].
- Support leadership development, including management training and soft skills (i.e. diplomacy, negotiation and storytelling) [12].
- Build capacity, including formal training in technical skills, research and mentorship [12].
- Mentorship should be cultivated early in training, with greater investment in mentorship in the mid-career level, when women leaders are at greatest risk for leaving the talent pipeline. Mentorship should be gender responsive, with men and women, alike being equipped with the knowledge and tools to recognise gender specific challenges, in addition to providing guidance on career advancement, work-life balance and overall resilience [12].
- Develop networks create space for women to connect with women in the global health community-locally, nationally and internationally. Spaces are needed where women can share their experiences unique to them allowing validation of their experiences and a place for both personal and professional development through peer-to-peer support [12].
- Increase flexibility for men and women in global health to accommodate personal, domestic, and family obligations, including increasing part-time opportunities and longer extensions [12].
- Improve policy and practice in terms of the health and safety risks women face in carrying out their health-related roles. This could include: increased health and emergency evacuation coverage; flexibility to accommodate for unexpected crises and personal emergencies; family support; and additional gender-sensitive security budgets [12].

1. Research and data should be disaggregated and reflexive in terms of sex and gender

Disaggregation of all health research, specifically accounting for sex and gender in the development of research questions, design experiments, analysis of data and reporting of results particularly as it pertains to health systems governance. Gender plays a significant role in considerations made in research design and data collection and affects outcomes on an individual, community and international level, as was evidenced with the quantitative and qualitative data collected during the course of research and ‘Improving women's health will require a serious and sustained investment in ‘big’ and better data, as well as in the collective and cumulative knowledge base of what is known as the ‘science of delivery’ [6, 7].

- Further research and analysis on the impact of women's leadership from design to implementation to health outcomes.
- Further research and analysis of the mid-career ‘pipe-line drain’ of women leaders in global health.

## Footnotes

† The notes appear after the main text.

## Notes

1 Women in Global Health (WGH) is an independent movement working with partners at all levels to achieve gender equity within global health leadership in order to achieve overall equity in global health. In order to address this disparity in leadership positions, Women in Global Health focuses on five strategic priority areas of: (1) Raising Awareness and Understanding; (2) Capacity Building; (3) Research and Data Collection; (4) Policy Analysis and Recommendations; and (5) Mentorship and Networking. WGH works with other global health gender-focused organizations and supporters to encourage stakeholders from governments, civil society, foundations, academia and professional associations and the private sector to achieve gender equality in global health leadership.

2 The Research in Gender and Ethics (RinGs) consortium is an initiative that brings together three health systems focused Research Programme Consortia (RPC): Future Health Systems, ReBUILD and RESYST, in a partnership to galvanise gender and ethics analysis in health systems. This partnership seeks to understand, and to encourage, a gendered and intersectional approach to the study of health systems care-seeking; financing and contracting; governance; and human resources for health by synthesising the current evidence base, stimulating new research and encouraging mutual learning and research uptake.

3 http://resyst.lshtm.ac.uk/rings
